# Phytochemicals and Immunomodulatory Effect of *Nelumbo nucifera* Flower Extracts on Human Macrophages

**DOI:** 10.3390/plants10102007

**Published:** 2021-09-24

**Authors:** Rungnapa Pankla Sranujit, Chanai Noysang, Patcharaporn Tippayawat, Nateelak Kooltheat, Thitiya Luetragoon, Kanchana Usuwanthim

**Affiliations:** 1 Faculty of Integrative Medicine, Rajamangala University of Technology Thanyaburi, Pathum Thani 12130, Thailand; chanai_n@rmutt.ac.th; 2 Center for Research and Development of Medical Diagnostic Laboratories, Faculty of Associated Medical Science, Khon Kaen University, Khon Kaen 40002, Thailand; patchatip@kku.ac.th; 3 Research Excellence Center for Innovation and Health Products, School of Allied Health Sciences, Walailak University, Nakhon Si Thammarat 80160, Thailand; nateelak.ko@wu.ac.th; 4 Cellular and Molecular Immunology Research Unit, Faculty of Allied Health Sciences, Naresuan University, Phitsanulok 65000, Thailand; thitiyal59@nu.ac.th (T.L.); kanchanau@nu.ac.th (K.U.)

**Keywords:** *Nelumbo nucifera*, lotus flower, phytochemicals, immunomodulatory, anti-inflammatory

## Abstract

This research characterizes phytochemicals inherent in lotus flower and investigates the antioxidant and immunomodulatory activity of ethyl acetate (EA) and ethyl alcohol (ET) lotus petal extracts. In the experiment, human monocytes-derived macrophages were stimulated by lipopoly-saccharide to mimic bacteria-induced inflammation. The results showed that ferulic acid, couma-rin, and chlorogenic acid were three dominant polyphenols. The EA and ET lotus petal extracts also possessed high antioxidant capability. Furthermore, the extracts exhibited immunomodulatory properties by suppressing TNF-α secretion in inflammatory-induced human macrophages by in-hibiting NF-κB-dependent inflammatory response. In essence, the lotus petal extracts possess reme-dial attributes beneficial to individuals afflicted with declined immune functions.

## 1. Introduction

*Nelumbo nucifera*, or sacred lotus, is an aquatic plant of the Nelumbonaceae family native to tropical and subtropical zones of Asia [1]. All parts of the lotus, such as root, stem, leaf, flower, and seed, are edible [1] and widely used in Ayurveda and traditional medicines [2,3,4]. Extracts of lotus contain various phytochemicals, including phenolic acids, alkaloids, flavonoids, and steroids [2,3,4,5,6,7,8,9], which promote antioxidant [3,4,10], anti-inflammatory [11,12], antidiabetic [13], anti-obesity [14], and anticancer [15] activities. These biological activities are beneficial for individuals with declined immune functions, especially those of advanced age [16,17], and immunosuppression in diabetic patients [18,19]. Despite herbal remedial benefits of every part of lotus, existing studies are nevertheless focused mainly on lotus leaf, seed, and root, while research on bioactivities and benefits of lotus flower is very limited.

The aim of this research is thus to investigate the phytochemical composition of lotus flower extracts and their antioxidant and anti-inflammatory activities. In the study, lotus petals were extracted by Soxhlet extractor using ethyl acetate and ethyl alcohol. In phytochemicals analysis, lotus petal extracts were screened by high-performance thin-layer chromatography (HPTLC) and sequentially identified by high-performance liquid chromatography with diode array detector (HPLC-DAD). Antioxidant activity was determined by 2,2-diphenyl-1-picrylhydrazyl (DPPH) free radical scavenging assay, and anti-inflammatory activity by levels of secretion of proinflammatory cytokines of human macrophages treated with lotus petal extracts. This research is expected to offer insight into immunomodulatory activity of lotus flower extracts and their potential use for treating individuals with declined immune functions.

## 2. Results

### 2.1. Lotus Petal Extracts

Prior to extraction, lotus petals were oven-dried at 60 °C for 48 h and the yield was 14.29% of fresh lotus petals. Ethyl acetate (EA) and ethyl alcohol (ET) lotus petal extracts were of yellow-brown viscous substances, with the extraction yields of 4.20% and 40.67% of dried lotus petal, respectively.

### 2.2. HPTLC Analysis of Phytochemical Composition of Lotus Petal Extracts

In HPTLC analysis, three phytochemical groups in lotus petal extracts: alkaloids, steroids, and phenolics, were analyzed under white light and 366 nm ultraviolet light. Table 1 tabulates the phytochemicals present in EA and ET lotus petal extracts, and Figures S1–S4, respectively, illustrate the HPTLC chromatograms of alkaloids, steroids, and phenolics. The analysis revealed that the phytochemical profile of EA lotus petal extract closely resembled that of ET extract.

### 2.3. HPLC Analysis of Phytochemical Composition of Lotus Petal Extracts

In HPLC-DAD analysis, six phytocompounds were used as standard phenolic acid and lactone compounds, including chlorogenic acid, rutin, ferulic acid, coumarin, quercetin, and kaempferol. Figure S5 illustrates the structures of standard phytocompounds. Comparing the spectral data at 280, 320, and 360 nm of the sample peaks with those obtained for the phenolic acid and lactone standards confirmed the reliability of qualification. Reliability of the method was further addressed by recovery and repeatability tests.

In Figure S6, the retention times of the standard phytocompounds were 4.39 ± 0.03, 6.02 ± 0.02, 7.08 ± 0.01, 10.89 ± 0.02, 12.29 ± 0.02, and 14.88 ± 0.00 min, respectively, with the corresponding maximum absorbance of 320, 360, 320, 280, 360, and 360 nm and the response factor of 74.22, 35.18, 199.56, 167.75, 33.82, and 71.57. Table S1 tabulates the parameters of HPLC-DAD analysis of the standard phytocompounds. Table 2 compares concentrations of phytocompounds in EA and ET lotus petal extracts, and Figures S7 and S8, respectively, illustrate the HPLC-DAD chromatograms of EA and ET lotus petal extracts. The analysis showed that both extracts contained all six phytochemicals of varying concentrations.

### 2.4. Total Phenolic Content of Lotus Petal Extracts

**Figure 1:**
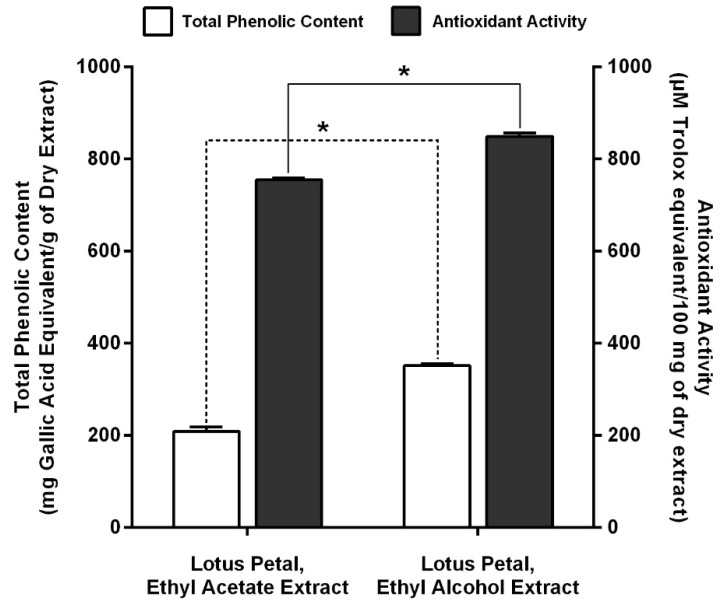
Total phenolic content and antioxidant activity of EA and ET lotus petal extracts, where * denotes statistical significance (*p* < 0.05).

In Figure 1, total phenolic contents of EA and ET lotus petal extracts determined by Folin-Ciocalteau method were 208.45 ± 10.11 and 351.08 ± 4.62 mg gallic acid equivalent/g dry extract, respectively.

### 2.5. Antioxidant Activity of Lotus Petal Extracts

Figure 1 also shows the antioxidant activity of EA and ET lotus petal extracts de-termined by DPPH radical scavenging assay. The antioxidant activity of EA and ET lotus petal extracts were 754.74 ± 4.44 and 849.49 ± 6.84 µM Trolox equivalent/100 mg dry extract, respectively.

### 2.6. Cellular Cytotoxicity of Lotus Petal Extracts

**Figure 2:**
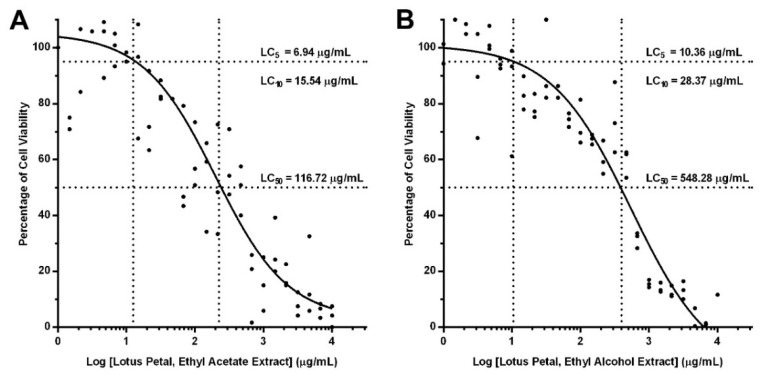
Dose-response curves of lotus petal extracts: (**A**) ethyl acetate extract, (**B**) ethyl alcohol extract. Human monocytes were treated with geometrically diluted lotus petal extracts, and median, 10%, and 5% lethal concentrations (LC_50_, LC_10_, and LC_5_) were determined from percentage of cell viability.

Figure 2A,B, respectively, illustrate the cellular cytotoxicity of EA and ET lotus petal extracts using dose-response curve fitting analysis. In the analysis, human monocytes were treated with geometrically diluted lotus petal extracts, and lethal concentrations (LC) of the extracts determined from percentage of cell viability. The median lethal concentration (LC50) of EA and ET lotus petal extracts were 116.72 and 548.28 µg/mL, respectively. In anti-inflammatory analysis, the concentrations of lotus flower extracts were 5% (LC5; low concentration) and 10% lethal concentrations (LC10; high concentration) to improve cell viability.

### 2.7. Anti-Inflammatory Activity of Lotus Petal Extracts

**Figure 3:**
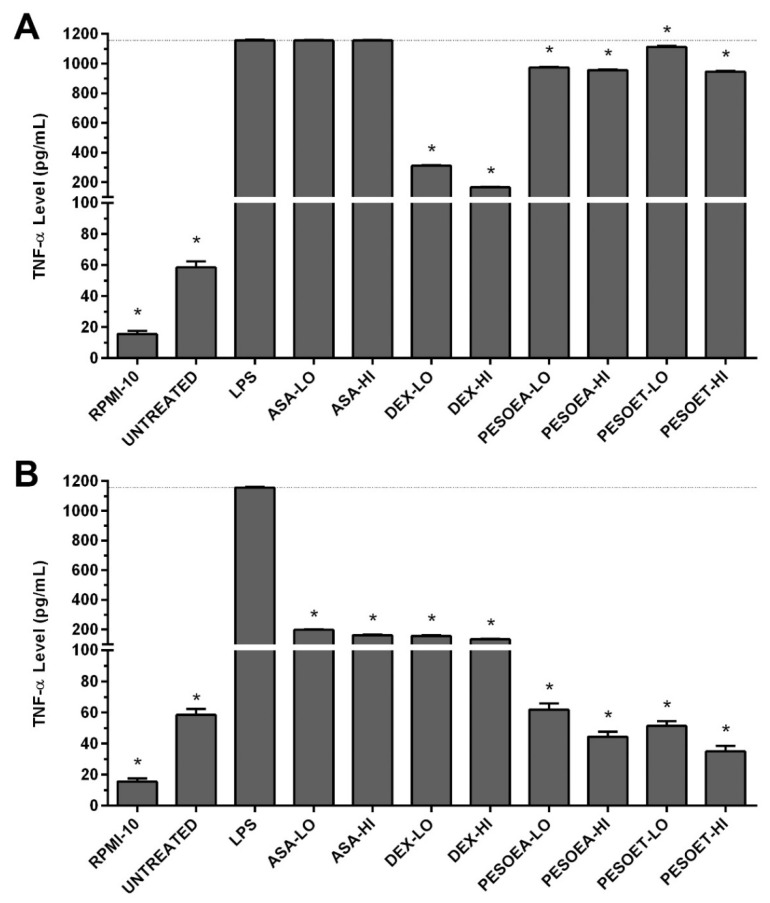
TNF-α level in human macrophages: (**A**) Pretreatment with lotus petal extracts for 6 h prior to LPS inflammatory stimulation for 6 h, (**B**) LPS stimulation for 6 h and post-treatment with lotus petal extracts for 6 h. Anti-inflammatory drugs (aspirin, ASA; dexamethasone, DEX) were tested along with the extracts. Levels of TNF-α were determined from cell culture supernatant using ELISA. RPMI-10 is culture medium, PE stands for petal extract, SO for Soxhlet extraction, EA for ethyl acetate, ET for ethyl alcohol, LO for low lethal concentration (5%), HI for high lethal concentration (10%), and * denotes *p* < 0.05.

The anti-inflammatory activity of lotus petal extracts was assessed by TNF-α secretion levels of human macrophages treated with lotus petal extracts. Specifically, lipopolysaccharide (LPS) was used to stimulate inflammatory response of the macro-phages, and levels of proinflammatory cytokine TNF-α were determined from cell cul-ture supernatant by enzyme-linked immunosorbent assay (ELISA). In Figure 3, the TNF-α level of untreated macrophages (control) was 58.47 ± 1.93 pg/mL and increased signif-icantly when stimulated by LPS to 1156.73 ± 4.14 pg/mL (*p* < 0.05), compared with un-treated cells.

In Figure 3A, pretreating human macrophages with lotus petal extracts prior to LPS inflammatory stimulation significantly decreased the TNF-α secretion (*p* < 0.05), compared with LPS-stimulated cells. Specifically, the TNF-α levels of human macro-phages pretreated with 5% (low) and 10% (high) concentrations of EA lotus petal extract significantly decreased to 973.46 ± 3.25 and 955.23 ± 4.79 pg/mL (*p* < 0.05), respectively, and those pretreated with low and high concentrations of ET extract also significantly decreased to 1112.44 ± 5.97 and 945.42 ± 4.33 pg/mL (*p* < 0.05), compared with LPS-stimulated cells. Nevertheless, pretreating macrophages with dexamethasone (DEX) was more effective in suppressing TNF-α secretion.

In Figure 3B, post-treating human macrophages with lotus petal extracts subsequent to LPS inflammatory stimulation significantly lowered the secretion of TNF-α when compared with LPS-stimulated cells (*p* < 0.05). The TNF-α levels of human macro-phages post-treated with 5% (low) and 10% (high) concentrations of EA lotus petal extract significantly decreased to 61.94 ± 3.88 and 44.44 ± 3.21 pg/mL (*p* < 0.05), respectively, and those post-treated with low and high concentrations of ET extract also significantly decreased to 51.41 ± 3.04 and 34.96 ± 3.63 pg/mL (*p* < 0.05), compared with LPS-stimulated cells. By comparison, when applied after exposure to the stimulant, EA and ET lotus petal extracts were more effective than aspirin (ASA) and dexamethasone (DEX) in suppressing TNF-α secretion.

## 3. Discussion

The experiment showed that ethyl alcohol (ET) achieved higher extractability than ethyl acetate (EA), with the extract yields of 40.67% and 4.20% of dried lotus petal, re-spectively. The phytochemicals present in the lotus petal extracts include chlorogenic acid, rutin, ferulic acid, coumarin, quercetin, and kaempferol. In addition, total phenolic content and antioxidant activity of ET lotus petal extract were higher than those of EA extract, while both extracts effectively suppressed TNF-α secretion of inflammatory-induced human macrophages, especially under the post-treatment condition.

In this research, quercetin and kaempferol were detected in EA and ET lotus petal extracts, similar to [2], which reported both polyphenols in lotus flower extracts. Never-theless, similar to this research, alkaloids, flavonoids, and steroids were reported in ex-tracts of lotus leaves [4,6,8], seed [2,9], embryo/plumule [2,7], and root [3]. Importantly, no previous research documented the presence of chlorogenic acid, ferulic acid, and coumarin in extracts of lotus parts [2,3,4,5,6,7,8,9].

Total phenolic content of EA and ET lotus petal extracts is positively correlated to solvent polarity gradient [20,21]. In addition, levels of antioxidant activity were positively correlated to total phenolic content (Figure 1), suggesting that greater total phenolic content contributed to higher antioxidant activity, consistent with [3,4,10]. Specifically, the antioxidant activity of ET lotus petal extract was 1.5 times higher than that of EA extract. In fact, DPPH assay, although straightforward and efficient, sometimes fails to account for certain antioxidant activities in plant extracts due to the complex structure of phytochemicals [4,5,22].

Despite numerous biological activities of phytochemicals in medicinal plants, use in high doses could be toxic [23,24]. Thus, to characterize cellular cytotoxicity of lotus petal extracts, EA and ET lotus petal extracts were tested on human monocytes in variable concentrations. In Figure 2, LC50 of ET lotus petal extract (548.28 µg/mL) was almost five times higher than that of EA extract (116.72 µg/mL), indicating substantially lower cellular cytotoxicity of ET extract. Nonetheless, this research utilized 5% (LC5) and 10% (LC10) concentrations of lotus petal extracts to improve cell viability.

Moreover, this research mimicked inflammatory response to bacterial infection in vitro by stimulating human macrophages with LPS, a strong stimulant specific to Toll-like receptor-4 (TLR4) [25], to induce NF-κB-dependent proinflammatory genes and cytokines expressions [26]. The results showed that treating macrophages with lotus petal extracts effectively suppressed TNF-α secretion (Figure 3), consistent with [11,12]. Specifically, pretreating human macrophages with EA and ET lotus petal extracts prior to LPS inflammatory stimulation decreased TNF-α secretion by 17% and 11%, respectively (Figure 3A). Meanwhile, post-treating human macrophages with EA and ET lotus petal extracts substantially decreased TNF-α secretion by 95% and 96%, respectively (Figure 3B). The findings indicated that lotus petal extracts are more effective in inflammation treatment (i.e., post-treatment) than prevention (pretreatment). Nonetheless, the anti-inflammatory activity of EA and ET lotus petal extracts on interleukin 6 (IL-6) was in-conclusive (Figure S9).

In light of the existence of publications on inhibition of NF-κB inflammatory pathway, this research did not investigate the signaling pathways of inhibitory activity. Specifically, inhibition of the NF-κB inflammatory pathway was attributable to chlorogenic acid [27], rutin [28], ferulic acid [29], coumarin [30], quercetin [31], and kaempferol [32]. In addition to anti-inflammatory activity, lotus flower extracts also exhibited antidiabetic [13], antiobesity [14], anticancer [15], and anti-HIV activities [6].

In this research, immunomodulatory effects of lotus petal extracts were investigated in innate immune system. Nonetheless, subsequent research would involve adaptive immune system in enhancing tumor cytotoxicity. In fact, organic phytochemicals inherent in lotus flowers possess high potential as ingredients in dietary supplements for immunomodulation in individuals with declined immune functions.

## 4. Materials and Methods 

### 4.1. Preparation of Plant Materials

Lotus flowers of *Nelumbo nucifera* species were acquired from a lotus plantation in Thailand’s Pathum Thani province. The species of the lotus was authenticated by Dr. Chanai Noysang (Thai Traditional Medicine College, Rajamangala University of Tech-nology Thanyaburi, Pathum Thani, Thailand). The lotus flowers were washed, and pet-als gently plucked. The lotus petals were then oven-dried at 60 °C for 48 h, ground, and sifted through 60-mesh sieve prior to storage in airtight container for extraction.

### 4.2. Extraction of Plant Materials

Pulverized lotus petal was extracted by Soxhlet extractor using ethyl acetate (EA) and ethyl alcohol (ET), whereby 15 g of ground lotus petal in extraction thimble was extracted by 300 mL of EA or ET for 8–10 h [33]. Coarse sediments were filtered and removed using cotton pads, and the extracts were refiltered with 11 µm cellulose filter (Whatman No. 1, Merck KGaA, Darmstadt, Germany). The extracts were subsequently dried using rotary evaporator (Hei-VAP Platinum 2, Heidolph Instruments GmbH & CO. KG, Schwabach, Germany) and stored in airtight container for further analysis.

### 4.3. Phytochemical Analysis by High-Performance Thin-Layer Chromatography (HPTLC)

In HPTLC analysis, standard phytochemicals were dissolved in absolute ethyl alcohol to a concentration of 1 mg/mL, including chlorogenic acid, ferulic acid, β-sitosterol, rutin (Acros Organics, Morris, NJ, USA), quercetin (Merck KGaA, Darmstadt, Germany), catechin, gallic acid, kaempferol, neferine (Sigma-Aldrich, Saint Louis, MO, USA), and caffeic acid (Dr. Ehrenstorfer GmbH, Augsburg, Germany). All chemicals and reagents used were of analytical grade and were purchased from Merck chemicals, Thailand. Figure S5 illustrates the structures and CAS registry numbers of standard phytochemicals. In addition, EA lotus petal extract was dissolved to a concentration of 10 mg/mL in 1:3 of ethyl acetate:methyl alcohol, and so it became ET lotus petal extract in absolute ethyl alcohol.

In addition, precoated silica gel aluminum plate 60F-254 (20 cm × 10 cm with 0.2 mm thickness) was used as stationary phase (E. Merck, Darmstadt, Germany). The lotus petal extracts were spotted on the HPTLC stationary phase alongside standard phytochemicals, and then separated by chromatography in the chamber of mobile phase. Mobile phase gradients were varied according to phytochemicals. Specifically, toluene:ethyl acetate:diethylamine (6:3:1 *v*/*v*/*v*) was used as mobile phase for alkaloids analysis, and dichloromethane:ethyl acetate:methyl alcohol:formic acid (6:2:1:1 *v*/*v*/*v*) was used for analysis of steroids. Meanwhile, dichloromethane:ethyl acetate:methyl alcohol:formic acid (25:10:7:8 *v*/*v*/*v* or 25:12:7:6 *v*/*v*/*v*) was used for analysis of phenolic acid and lactone compounds. Separated samples of EA and ET extracts and standard phytochemicals were developed by detection reagent and visualized by white light and ultraviolet light at 254 and 366 nm wavelengths using a CAMAG TLC scanner for densitometric evaluation of chromatograms (Muttenz, Switzerland). Specifically, alkaloids were detected with Dragendorff’s reagent, steroids with 10% sulfuric acid in ethyl alcohol, phenolics with 1% aluminum chloride in ethyl alcohol, or 2% ferric chloride in methyl alcohol. The retention factor on HPTLC plate was determined and compared against phenolic acid and lactone standards.

### 4.4. Phytochemical Analysis by High-Performance Liquid Chromatography with Diode Array Detector (HPLC-DAD)

In HPLC-DAD analysis, standard phenolics were first prepared by dissolving in 70% methyl alcohol to a concentration of 20 mg/mL, including chlorogenic acid, rutin, ferulic acid (Acros Organics, Morris, NJ, USA), coumarin (ChromaDex, Inc., Irvine, CA, USA), kaempferol (Sigma-Aldrich, Saint Louis, MO, USA), and quercetin (Merck KGaA, Darmstadt, Germany). The standard phenolics were further diluted to 0.5, 1, 3, 5, 10, 50, 100, and 500 µg/mL for the purpose of rendering standard curves. In addition, standard phenolics at 2, 20, and 200 µg/mL concentrations were used to validate the HPLC results. 

Lotus petal extracts were then prepared by dissolving in 70% methyl alcohol to a concentration of 10 mg/mL, vortexed, and centrifuged at 3000 rpm for 5 min. The extracts were filtered with 0.45 µm cellulose acetate syringe filter to remove coarse particles and aliquoted into microcentrifuge tubes for HPLC analysis. 

In HPLC-DAD analysis, samples of standard phenolics and EA and ET extracts were separated by liquid chromatography (Agilent 1260 Infinity, Agilent Technologies, Santa Clara, CA, USA) with C18 stationary phase column (ZORBAX Eclipse Plus, Agilent Technologies, Santa Clara, CA, USA), with 4.6 and 100 mm in diameter and length packed with 5 µm silica gel. The samples (20 µL each) were then injected into HPLC, and the analysis carried out at 30 °C using variable mixtures of acetonitrile (ACN) and 0.1% trifluoroacetic acid in water (TFA) (Fisher Scientific UK Ltd., Loughborough, UK) as mobile phase. The mobile-phase gradients in a cycle of 20 min were 10% (1 min), 20% (2 min), 25% (3 min), 30% (2 min), 35% (3 min), 100% (6 min), and 10% (3 min) of ACN. Absorbance of the separated samples was measured by diode array detector (G1315D—1260 DAD VL, Agilent Technologies, Santa Clara, CA, USA). The concentrations of phytochemicals were determined according to the response factors of phenolic acid and lactone standards.

### 4.5. Determination of Total Phenolic Content

Total phenolic contents of lotus petal extracts were determined by Folin-Ciocalteau method [34] using a 96-well plate. Dried EA and ET lotus petal extracts were first dissolved in dimethyl sulfoxide (DMSO) (Bio Basic Inc., Markham, ON, Canada) to a concentration of 10 mg/mL. In the analysis, 2.5 µL of lotus petal extracts was first diluted by 100 µL of deionized water and 5 µL of Folin-Ciocalteau reagent (Merck KGaA, Darmstadt, Germany) added prior to incubation at room temperature for 5 min with shaking. Then, 100 µL of 2% sodium carbonate solution was added and incubated at 50 °C for another 60 min. The absorbance at 750 nm was measured using microplate reader (PerkinElmer Inc., Waltham, MA, USA). Gallic acid (Sigma-Aldrich, Saint Louis, MO, USA) was used as standard phenolic. Total phenolic content was determined based on standard curve and expressed as mg Gallic acid equivalent/g dry extract.

### 4.6. Determination of Antioxidant Activity

Antioxidant activity of lotus petal extracts were determined by 2,2-diphenyl-1-picrylhydrazyl (DPPH) radical scavenging assay [35] using a 96-well plate. In the analysis, 5 µL of lotus petal extracts was mixed with 50 µL deionized water and 50 µL of 0.2 mM DPPH (Merck KGaA, Darmstadt, Germany) in methanol added prior to incubation at room temperature for 2 h in the dark with shaking. The absorbance at 517 nm was measured using a microplate reader (PerkinElmer Inc., Waltham, MA, USA). Trolox (Sigma-Aldrich, Saint Louis, MO, USA) was used as standard antioxidant. The antioxidant activity was determined based on standard curve and expressed as µM Trolox equivalent/100 mg dry extract.

### 4.7. Isolation of Human Peripheral Blood Monocytes

The isolation of human monocytes was approved by the Khon Kaen University Ethics Committee for Human Research, Khon Kaen University, Khon Kaen, Thailand, under certification number HE621066. Blood donors were healthy, had no history of illness with infectious diseases or being infected or inflamed due to microbial infections, had no history of autoimmune diseases, and had not been on antibiotics for a minimum period of four weeks [36]. Furthermore, peripheral blood was obtained with consent.

In monocytes isolation, donated blood was transferred to 50 mL centrifuge tubes and centrifuged at 3000 rpm for 30 min to separate buffy coat from red blood cells. The buffy coat was collected and resuspended with 20 mL of Hank’s balanced salt solution (HBSS). Using 15 mL centrifuge tube, 10 mL of diluted buffy coat was placed on top of 5 mL of 1.077 g/mL Lymphoprep density gradient solution (Alere Technologies AS, Oslo, Norway) and centrifuged at 3000 rpm for 30 min to separate peripheral blood mononuclear cells (PBMCs) from granulocytes. PBMCs were collected and resuspended with complete RPMI-1640 cell culture medium (RPMI-1640 medium with 10% fetal bovine serum and 1% penicillin/streptomycin antibiotic mixture) (Thermo Fisher Scientific, Waltham, MA, USA). 

Human monocytes were then isolated by density gradient centrifugation. Using 15 mL centrifuge tube, 5 mL of PBMCs suspension was placed on top of 10 mL of 1.068 g/mL Percoll gradient solution (GE Healthcare Bio-Sciences AB, Uppsala, Sweden). Monocytes were transferred to another 50 mL centrifuge tube, washed by resuspending in HBSS, and centrifuged at 2000 rpm for 10 min. Cells were further washed four times with sequential centrifugation of 1700, 1500, 1200, and 900 rpm for 10 min each to remove platelets. Isolated human monocytes were resuspended in complete cell culture medium and examined for cell viability by trypan blue exclusion test. Cells were cultured in complete RPMI medium for another seven days to allow monocytes to differentiate into monocytes-derived macrophages (MDMs).

### 4.8. Evaluation of Cellular Cytotoxicity by Neutral Red Uptake Assay

Cellular cytotoxicity of lotus petal extracts was evaluated by neutral red uptake assay [37], whereby 5 × 104 cells/well of isolated monocytes were plated in a 96-well plate (SPL Life Sciences Co., Ltd., Gyeonggi-do, Korea) and allowed to rest for 24 h at 37 °C and 5% CO_2_ in humidified incubator. Meanwhile, lotus petal extracts were diluted geometrically to concentrations of 1–10,000 µg/mL [38] with complete RPMI medium (Thermo Fisher Scientific, Waltham, MA, USA).

In cellular cytotoxicity assay, isolated monocytes were incubated in 100 µL of di-luted lotus petal extracts (1–10,000 µg/mL) for 12 h at 37 °C and 5% CO_2_ in humidified incubator. Cells were then washed with HBSS and incubated in 50 µg/mL neutral red dye (Merck KGaA, Darmstadt, Germany) in complete RPMI medium for another 2 h. Afterward, cells were washed three times with HBSS and lysed with acid alcohol solution (1% acetic acid in 50% ethyl alcohol) to dissolve neutral red dye in cells. Absorbance of the neutral red dye was measured at 545 nm using a microplate reader (PerkinElmer Inc., Waltham, MA, USA), and percentage of cell viability calculated. The viability of cells treated with lotus petal extracts was used to calculate median (LC50), 10% (LC10), and 5% lethal concentrations (LC5) of the extracts by dose-response curve fitting analysis using GraphPad Prism (GraphPad Software, Inc., La Jolla, CA, USA) [39]. LC5 and LC10 of EA and ET lotus petal extracts were subsequently used in assessing the anti-inflammatory activity. 

### 4.9. Investigation of Anti-Inflammatory Activity of Lotus Petal Extracts

The anti-inflammatory activity of lotus petal extracts was determined by levels of proinflammatory cytokine (tumor necrosis factor (TNF)-α) secretion in human macro-phages. In anti-inflammatory assessment, 2 × 105 cells/well were plated in a 24-well plate (SPL Life Sciences Co., Ltd., Gyeonggi-do, Korea) and allowed to rest for 24 h at 37 °C and 5% CO_2_ in humidified incubator. 

For baseline condition, cells were treated with 500 µL of 5% (low) and 10% (high) lethal concentrations of lotus petal extracts for 6 h. For pretreated condition, cells were treated with lotus petal extracts for 6 h, followed by stimulation of inflammatory response with 10 ng/mL LPS for 6 h. For post-treated condition, cells were first treated with 10 ng/mL LPS (Sigma-Aldrich, Saint Louis, MO, USA) for 6 h prior to treating with lotus petal extracts for 6 h. The concentrations of lotus petal extracts were 5% (LC5) and 10% (LC10) lethal concentrations.

Furthermore, as positive controls, aspirin and dexamethasone (i.e., nonsteroidal and steroidal anti-inflammatory drugs), at therapeutic concentration ranges of 5.42–10.83 µg/mL and 12.50–150 ng/mL, respectively [40,41,42], were tested alongside the lotus petal extracts for anti-inflammatory activity. Cell culture supernatant of LC5 and LC10 extracts were collected for evaluation of TNF-α secretion by ELISA.

### 4.10. Evaluation of Proinflammatory Cytokines Production by Enzyme-Linked Immuno-Sorbent Assay (ELISA)

Levels of proinflammatory cytokine (TNF-α) were determined by sandwich ELISA (BioLegend, San Diego, CA, USA) [43,44]. In the analysis, ELISA plates were first coated with 100 µL of capture antibody specific to TNF-α at 4 °C for 24 h, and then washed four times with phosphate buffer saline containing 0.1% Tween-20 (PBS-T). The plates were then blocked by assay diluent-A at room temperature for 1 h with shaking. They were then washed four times, and 100 µL of cell culture supernatant of LC5 and LC10 extracts was added prior to incubation at room temperature for 2 h with shaking. 

The plates were further washed four times, and 100 µL of diluted biotin-labelled detection antibody was added prior to incubation at room temperature for 1 h with shaking. Afterward, the plates were washed four more times, and 100 µL of avidin-HRP was added to each well before incubation at room temperature for 30 min in the dark with shaking. They were washed four more times, and 100 µL of TMB-substrate solution was added to each well and incubated at room temperature for 15 min in the dark. At termination, 100 µL of 1 M sulfuric acid was added to stop the reaction. Absorbance at 450 nm was measured with subtraction at 570 nm by microplate reader (PerkinElmer, Inc., Waltham, MA, USA). TNF-α standard graph was established, and levels of TNF-α were then determined.

### 4.11. Experimental Design and Statistical Analysis

Experiments were independently carried out in triplicate. Lethal concentrations were determined from viability of cells treated with lotus petal extracts using dose-response curve fitting analysis. Differences between total phenolic content and antioxidant activity of EA and ET lotus petal extracts were analyzed by *t*-test. Meanwhile, differences between levels of TNF-α of untreated and treated (with lotus petal extracts) human macrophages were determined by one-way analysis of variance (ANOVA). Statistical analysis was performed using GraphPad Prism (GraphPad Software, Inc., La Jolla, CA, USA) with the 95% confidence level (*p* < 0.05).

## 5. Conclusions

This research investigated the antioxidant and anti-inflammatory activity of ethyl acetate (EA) and ethyl alcohol (ET) lotus petal extracts. In anti-inflammation analysis, human monocytes-derived macrophages were stimulated by lipopolysaccharide to mimic bacteria-induced inflammation. The experimental results revealed three dominant phytocompounds in EA and ET lotus petal extracts: chlorogenic acid, ferulic acid, and coumarin. In addition, EA and ET extracts possess high free-radical scavenging capacity. The extracts also exhibited immunomodulatory properties through suppression of TNF-α levels by inhibiting NF-κB-dependent inflammatory response. In essence, the lotus petal extracts hold great potential for further development into dietary supplements beneficial for those suffering declining immune functions.

## Figures and Tables

**Table 1:** Phytochemical composition of EA and ET lotus petal extracts based on HPTLC chromatograms.

Phytochemical	Lotus Petal Extract	
	Ethyl Acetate	Ethyl Alcohol
**Alkaloids**		
Neferine	−	−
Other	+	+
**Steroids**		
Β-sitosterol	+/−	+/−
Others	+	+
**Phenolics**		
Quercetin	+	+
Kaempferol	+	+
Chlorogenic Acid	−	−
Rutin	−	−
Caffeic Acid	+/−	−
Gallic Acid	−	−
Catechin	−	−
Ferulic Acid	−	−
Others	+	+

+, −, and +/−, respectively, denote detectable colorimetric or fluorescence reactions, no detectable reaction, and unclear reaction.

**Table 2:** Phytochemical composition of lotus petal extracts based on HPLC-DAD chromatograms.

Phytochemical (µg/mL)	Lotus Petal Extract	
	Ethyl Acetate	Ethyl Alcohol
Chlorogenic Acid	1.45 ± 0.120	3.10 ± 1.070
Rutin	2.42 ± 0.020	5.61 ± 3.150
Ferulic Acid	20.62 ± 1.560	51.27 ± 1.190
Coumarin	1.72 ± 0.330	4.61 ± 0.590
Quercetin	43.34 ± 0.280	25.95 ± 0.730
Kaempferol	92.17 ± 0.850	31.84 ± 1.810

## Supplementary Materials

The following are available online at https://www.mdpi.com/article/10.3390/plants10102007/s1, Figure S1: Thin-layer chromatograms of alkaloids in lotus petal extracts, Figure S2: Thin-layer chromatograms of steroids in lotus petal extracts, Figure S3: Thin-layer chromatograms of phenolics in lotus petal extracts by aluminum chloride solution, Figure S4: Thin-layer chromatograms of phenolics in lotus petal extracts by ferric chloride solution, Figure S5: Chemical structures of standard phytochemi-cals and CAS registry numbers, Figure S6: HPLC-DAD chromatograms of standard phytocom-pounds, Figure S7: HPLC-DAD chromatograms of ethyl acetate lotus petal extract, Figure S8: HPLC-DAD chromatograms of ethyl alcohol lotus petal extract, Figure S9: IL-6 level of human macrophages treated with lotus petal extracts, Table S1: Parameters of HPLC-DAD analysis of standard phenolics.
