# Supplementary material for: Phytochemicals and Immunomodulatory Effect of Nelumbo nucifera Flower Extracts on Human Macrophages
Source: Plants (Basel). 2021 Sep 24;10(10):2007. doi: 10.3390/plants10102007 (PMC8537166; doi:10.3390/plants10102007)
Supplement: Supplementary file 1 [file plants-10-02007-s001.zip › plants-1333330-supplementary.pdf]

Supplementary material

Phytochemicals and Immunomodulatory Effect of *Nelumbo nucifera* Flower Extracts on Human Macrophages

Rungnapa Pankla Sranujit [1,*], Chanai Noysang [1], Patcharaporn Tippayawat [2], Nateelak Kooltheat [3], Thitiya Luetragoon [4] and Kanchana Usuwanthim [4]

[1] Faculty of Integrative Medicine, Rajamangala University of Technology Thanyaburi, Pathum Thani 12130, Thailand; chanai_n@rmutt.ac.th

[2] Center for Research and Development of Medical Diagnostic Laboratories, Faculty of Associated Medical Science, Khon Kaen University, Khon Kaen 40002, Thailand; patchatip@kku.ac.th

[3] Research Excellence Center for Innovation and Health Products, School of Allied Health Sciences, Walailak University, Nakhon Si Thammarat 80160, Thailand; nateelak.ko@wu.ac.th

[4] Cellular and Molecular Immunology Research Unit, Faculty of Allied Health Sciences, Naresuan University, Phitsanulok 65000, Thailand; thitiyal59@nu.ac.th (T.L.); kanchanau@nu.ac.th (K.U.)

[*] Correspondence: rungnapa_s@rmutt.ac.th; Tel.: +66-2-592-1999 (ext. 1424); Fax: +66-2-592-1900

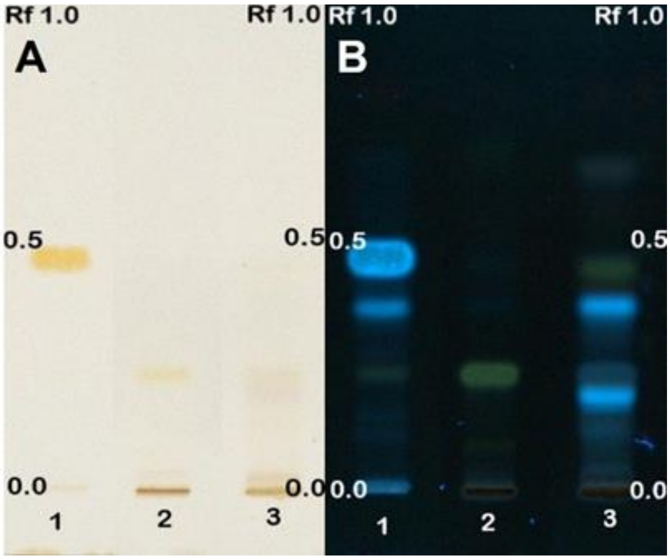

Figure S1. Thin-layer chromatograms of alkaloids in lotus petal extracts. Lotus petal extracts were spotted and separated by HPTLC using toluene:ethyl acetate:diethylamine (6:3:1, *v*/*v*/*v*) as mobile phase. HPTLC plate was then sprayed with Dragendorff's reagent and inspected under white light (**A**) and 366 nm (**B**). Tract: 1, neferine; 2, ethyl acetate extract of lotus petal; 3, ethyl alcohol extract of lotus petal.

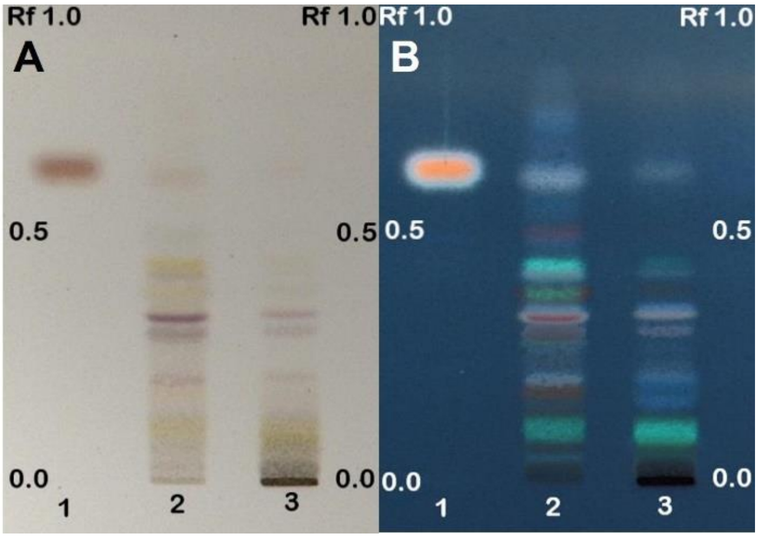

Figure S2. Thin-layer chromatograms of steroids in lotus petal extracts. Lotus petal extracts were spotted and separated by HPTLC using toluene:ethyl acetate:methanol:formic acid (6:2:1:1; *v*/*v*/*v*) as mobile phase. HPTLC plate was then sprayed with 10% sulfuric acid solution and inspected under white light (**A**) and 366 nm (**B**). Tract: 1, β-sitosterol; 2, ethyl acetate extract of lotus petal; 3, ethyl alcohol extract of lotus petal.

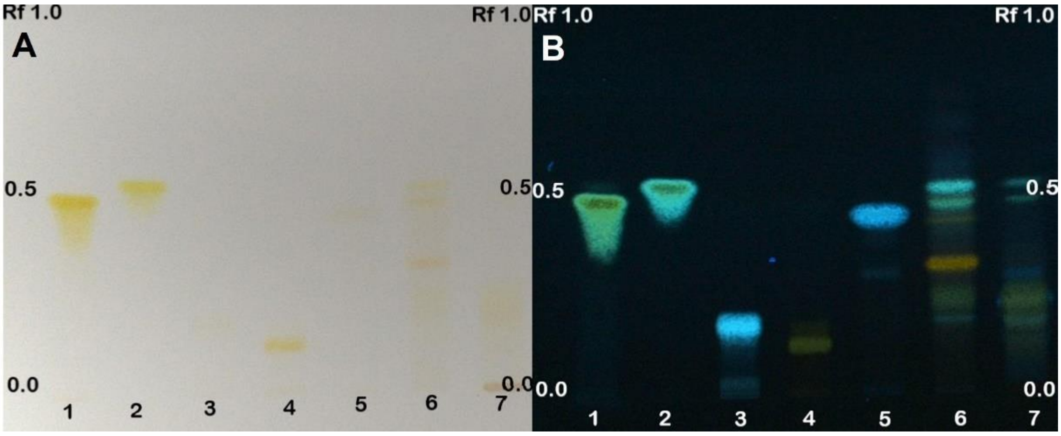

Figure S3. Thin-layer chromatograms of phenolics in lotus petal extracts by aluminum chloride solution. Lotus petal extracts were spotted and separated by HPTLC using toluene:ethyl acetate:methanol:formic acid (25:10:7:8; *v*/*v*/*v*) as mobile phase. HPTLC plate was then sprayed with 1% aluminum chloride solution and inspected under white light (**A**) and 366 nm (**B**). Tract: 1, quercetin; 2, kaempferol; 3, chlorogenic acid; 4, rutin; 5, caffeic acid; 6, ethyl acetate extract of lotus petal; 7, ethyl alcohol extract of lotus petal.

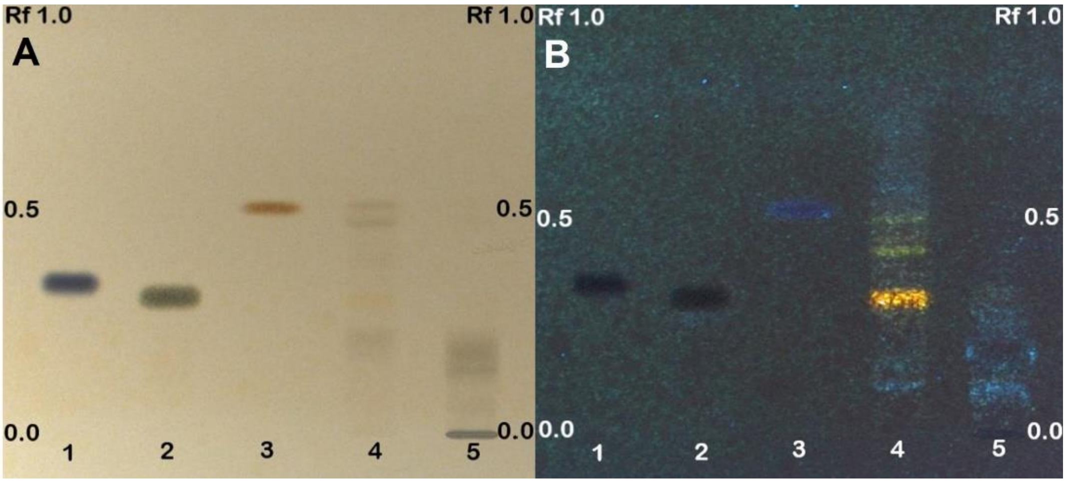

Figure S4. Thin-layer chromatograms of phenolics in lotus petal extracts by ferric chloride solution. Lotus petal extracts were spotted and separated by HPTLC using toluene:ethyl acetate:methanol:formic acid (25:12:7:6; *v*/*v*/*v*) as mobile phase. HPTLC plate was then sprayed with 2% ferric chloride solution and inspected under white light (**A**) and 366 nm (**B**). Tract: 1, gallic acid; 2, catechin; 3, ferulic acid; 4, ethyl acetate extract of lotus petal; 5, ethyl alcohol extract of lotus petal.

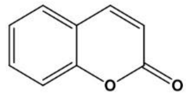

Coumarin [91-64-5]

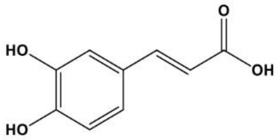

Caffeic Acid [331-39-5]

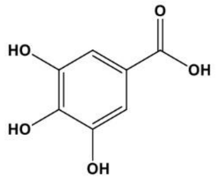

Gallic Acid [5995-86-8]

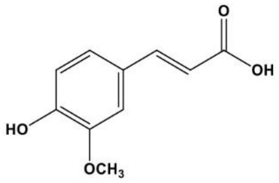

Ferulic Acid [1135-24-6]

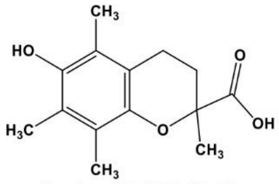

Trolox [53188-07-1]

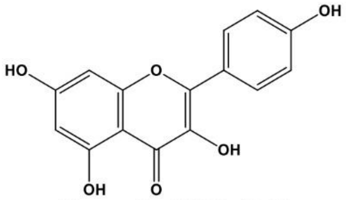

Kaempferol [520-18-3]

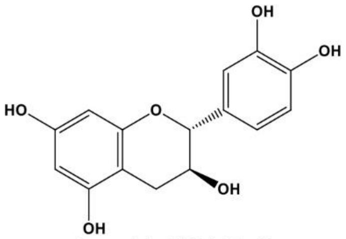

Catechin [154-23-4]

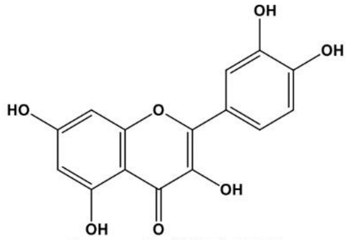

Quercetin [6151-25-3]

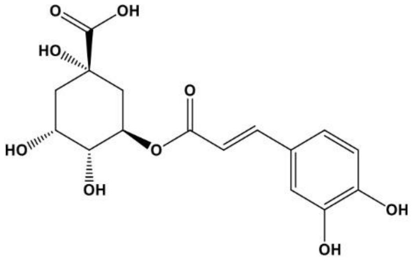

Chlorogenic Acid [327-97-9]

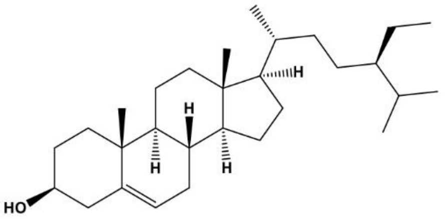

β-sitosterol [83-46-5]

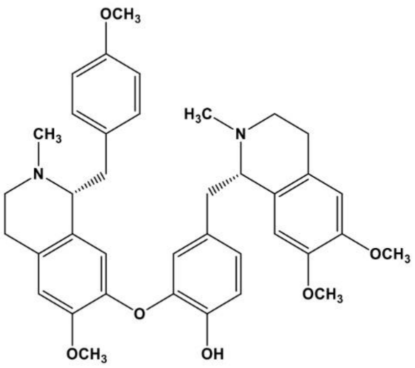

Neferine [2292-16-2]

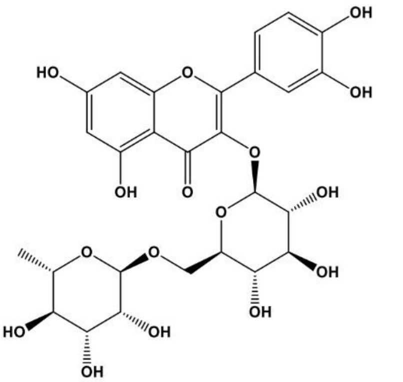

Rutin [153-18-4]

Figure S5. Chemical structures of standard phytochemicals and CAS registry numbers.

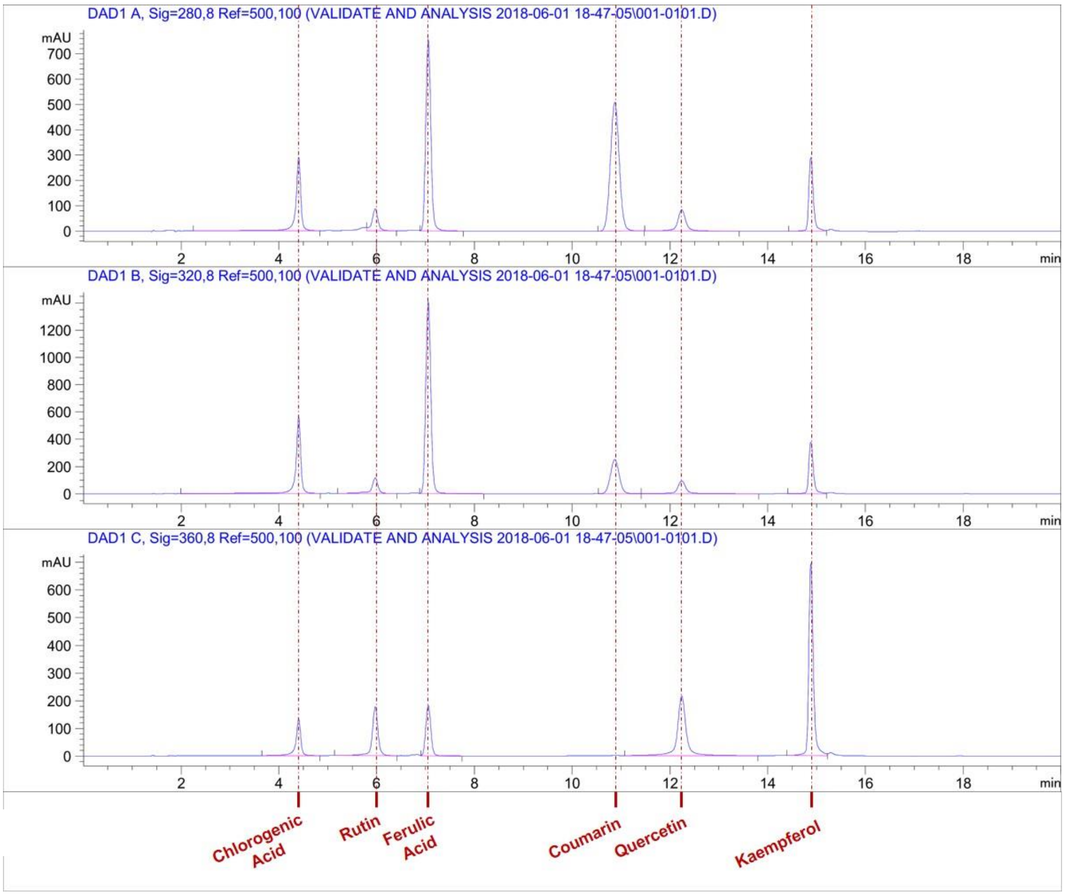

Figure S6. HPLC-DAD chromatograms of standard phytocompounds. The standard phenolics were separated through C18 stationary phase column by high-performance liquid chromatography using a gradient mobile phase of acetonitrile and 0.1% trifluoroacetic acid in water with a flow rate of 0.8 mm/min, 20 min. Absorbances of each phytochemical was then measured by diode array detector at 280 (**A**), 320 (**B**), and 360 (**C**) nm. Analysis by Agilent 1260 Infinity has limit of detection (LOD) of 25 ppt and limit of quantitation (LOQ) of 50 ppt. From left to right: chlorogenic acid, rutin, ferulic acid, coumarin, quercetin, and kaempferol.

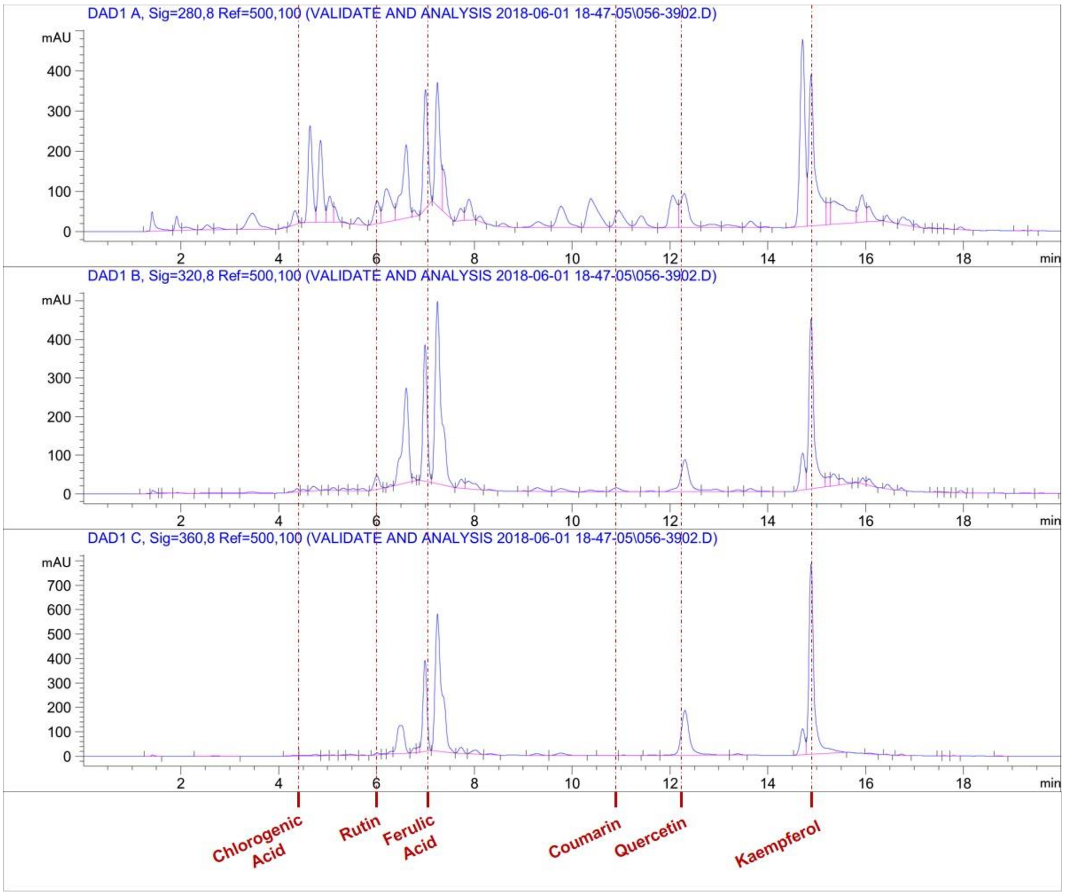

Figure S7. HPLC-DAD chromatograms of ethyl acetate extract of lotus petal by Soxhlet. The extract was separated through C18 stationary phase column by high-performance liquid chromatography using a gradient mobile phase of ac-etonitrile and 0.1% trifluoroacetic acid in water with a flow rate of 0.8 mm/min, 20 min. Absorbances of each phytochem-ical was then measured by diode array detector at 280 (**A**), 320 (**B**), and 360 (**C**) nm.

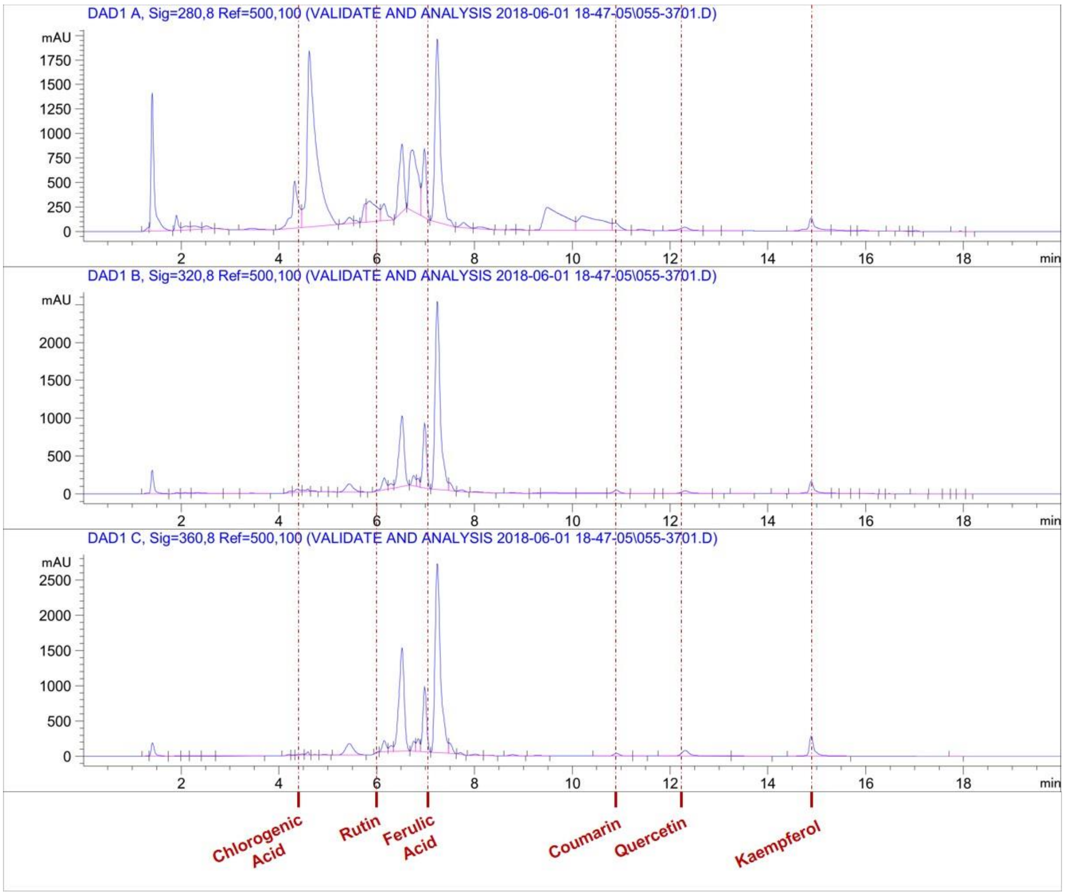

Figure S8. HPLC-DAD chromatograms of ethyl alcohol extract of lotus petal by Soxhlet. The extract was separated through C18 stationary phase column by high-performance liquid chromatography using a gradient mobile phase of ac-etonitrile and 0.1% trifluoroacetic acid in water with a flow rate of 0.8 mm/min, 20 min. Absorbances of each phytochem-ical was then measured by diode array detector at 280 (**A**), 320 (**B**), and 360 (**C**) nm.

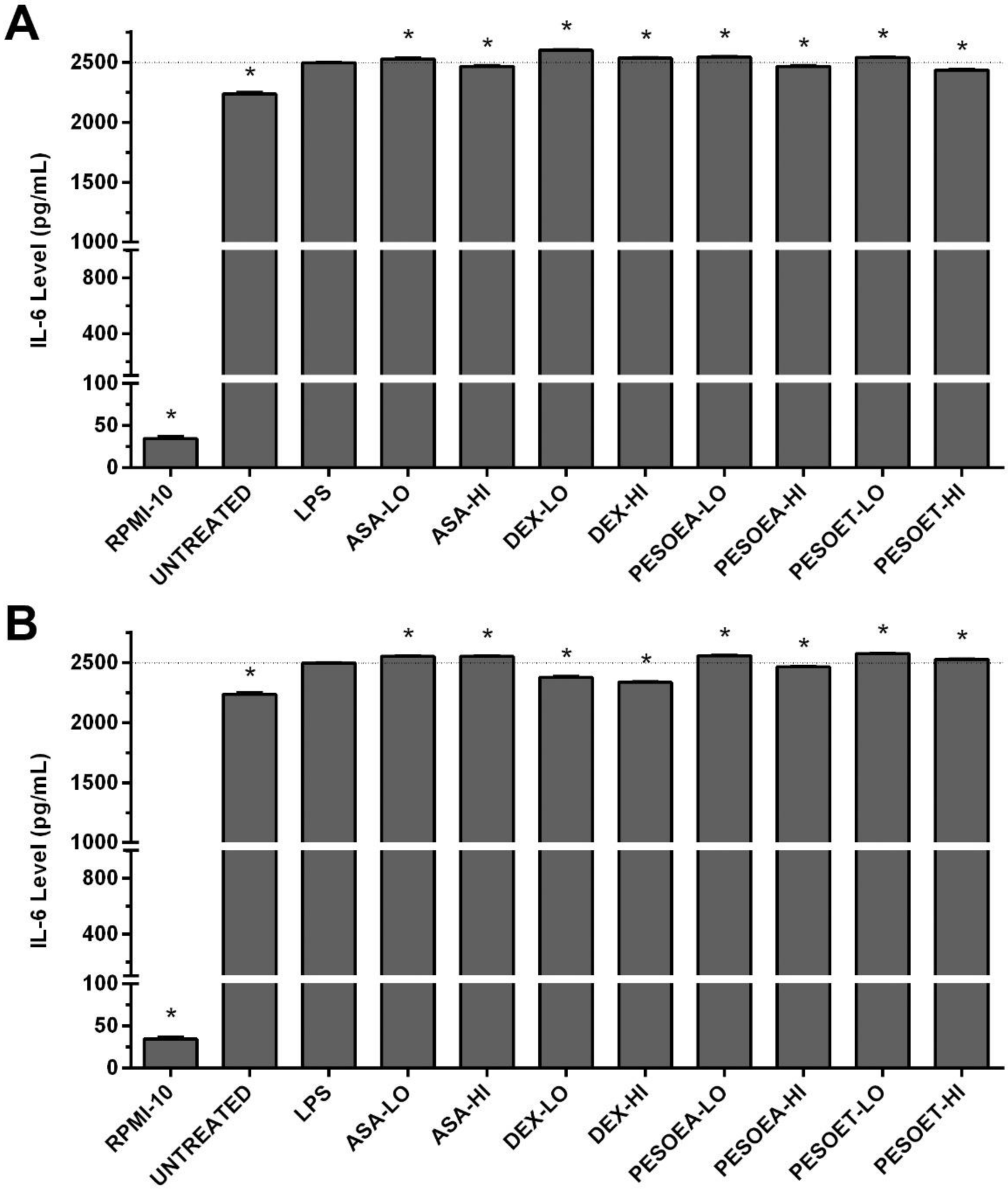

Figure S9. IL-6 level of human macrophages treated with lotus petal extracts: (**A**) human macrophages were pre-treated with lotus petal extracts for 6 h prior to LPS inflammatory response stimulation for 6 h; (**B**) human macrophages were stimulated with LPS for 6 h and post-treated with lotus flower extracts for 6 h. Anti-inflammatory drugs (aspirin, ASA; dexamethasone, DEX) were tested alongside the lotus petal extracts. Cell culture supernatant was collected and levels of IL-6 measured by ELISA. RPMI-10 is culture medium, PE stands for petal extract; SO for Soxhlet extraction; EA for ethyl acetate; ET for ethyl alcohol; LO for low concentration (LC5); HI for high concentration (LC10); and * denotes $p < 0.05$ compared with LPS-stimulated cells.

Table S1. Parameters of HPLC-DAD analysis of standard phenolics.

	Chlorogenic	Rutin	Ferulic Acid	Coumarin	Quercetin	Kaempferol
			HPLC Parameter			
**Retention Time (min)**	4.39 ± 0.03	6.02 ± 0.02	7.08 ± 0.01	10.89 ± 0.02	12.29 ± 0.02	14.88 ± 0.00
**Max. Absorbance (nm)**	320	360	320	280	360	360
			Linear Regression Parameter			
**Slope**	50.018×	34.514×	77.932×	95.850×	66.969×	58.696×
**Y-intercept**	191.84	−32.12	590.81	373.42	−732.27	274.12
**Correlation Coeff.**	0.9967	0.9961	0.9961	0.9983	0.9983	0.9978
			Area Under Curve (mAU*s), Peak Area			
**Concentration 50 µg/mL**	3700.76	1763.99	9986.57	8400.27	1677.68	3528.32
	3725.71	1757.61	9969.71	8382.80	1703.12	3598.90
	3706.45	1754.82	9977.78	8378.85	1691.72	3607.96
**Response Factor**	74.22	35.18	199.56	167.75	33.82	71.57
